# Stimulation of Cultured H9 Human Embryonic Stem Cells with Thyroid Stimulating Hormone Does Not Lead to Formation of Thyroid-Like Cells

**DOI:** 10.1155/2012/634914

**Published:** 2012-03-11

**Authors:** Mykola I. Onyshchenko, Igor G. Panyutin, Irina V. Panyutin, Ronald D. Neumann

**Affiliations:** ^1^Imaging Sciences Training Program, Clinical Center and National Institute of Biomedical Imaging and Bioengineering, National Institutes of Health, Bethesda, MD 20892, USA; ^2^Department of Radiology and Imaging Sciences, Clinical Center, National Institutes of Health, Bethesda, MD 20892, USA

## Abstract

The sodium-iodine symporter (NIS) is expressed on the cell membrane of many thyroid cancer cells, and is responsible for the radioactive iodine accumulation. However, treatment of anaplastic thyroid cancer is ineffective due to the low expression of NIS on cell membranes of these tumor cells. Human embryonic stem cells (ESCs) provide a potential vehicle to study the mechanisms of NIS expression regulation during differentiation. Human ESCs were maintained on feeder-independent culture conditions. RT-qPCR and immunocytochemistry were used to study differentiation marker expression, ^125^I uptake to study NIS function. We designed a two-step protocol for human ESC differentiation into thyroid-like cells, as was previously done for mouse embryonic stem cells. First, we obtained definitive endoderm from human ESCs. Second, we directed differentiation of definitive endoderm cells into thyroid-like cells using various factors, with thyroid stimulating hormone (TSH) as the main differentiating factor. Expression of pluripotency, endoderm and thyroid markers and ^125^I uptake were monitored throughout the differentiation steps. These approaches did not result in efficient induction of thyroid-like cells. We conclude that differentiation of human ESCs into thyroid cells cannot be induced by TSH media supplementation alone and most likely involves complicated developmental patterns that are yet to be understood.

## 1. Background

Human ESCs are the subject of extremely intensive research nowadays. ESCs possess the ability to differentiate into all cell types, and further form all tissues of the body. This unique feature makes these cells a valuable source for *in vitro* formation of differentiated cells which can be used in clinical applications, mostly in substitutive therapy [1]. Embryonic stem cells can also provide an important tool for developmental biology studies. This is especially true for human embryo development studies, where few approved human ESC lines can be used for the research and numerous ethical issues exist [2, 3].

Differentiation of ES cells to thyroid cells has been proposed as an approach to study thyroid gland development where mechanisms still remain unclear; especially concerning the early development stages [4, 5]. Human ES cells possess considerable self-renewing potential and could, in theory, provide an unlimited source of derived thyroid cells. Such substitutive therapy may have clinical application in diseases such as congenital hypothyroidism, thyroid cancer, and acquired hypothyroidism from autoimmune thyroiditis [4, 5].

Thyroid tissue cells originate from all three embryonic germ layers. The stroma of the thyroid gland develops from mesoderm; parafollicular cells (C-cells), representing calcitonin secreting tissue within the thyroid gland, originate from neural crest (derivative of ectoderm); and thyroid follicular cells, the most abundant cellular population, are from endodermal origin. Follicular cells possess the unique ability to absorb and concentrate molecular iodine, the crucial element of thyroid hormones (triiodothyronine [T3] and thyroxine [T4]). This phenomenon provides the molecular mechanism for diagnostics and treatment of the variety of thyroid gland diseases with radioactive iodine isotopes, mostly with ^131^I.

We implemented an approach where human ESCs were used as a model for studying the mechanisms of NIS maturation during their differentiation to thyroid follicular cells. Dedifferentiation takes place in cancer cells, and it is especially prominent in high-grade tumors. Therefore, studying the reverse process, differentiation, may reveal the patterns of NIS expression regulation. This knowledge may provide alternative ideas of NIS induction in cancer cells and could open new possibilities for radioisotope treatment of thyroid cancer.

Previous studies on mouse ESCs demonstrated their successful differentiation into thyroid cells with thyroid stimulating hormone (TSH) as the main differentiating factor [5, 6]. We tested various approaches to produce thyroid follicular cells from human ESCs including that used for differentiation of mouse ESCs. We used both a one step protocol (direct differentiation from human ESCs to thyroid-like cells) and a two-step protocol through endoderm formation. However, our attempts did not result in an efficient induction of thyroid follicular cells. We attributed this to the differences in the differentiation patterns in mice and humans [7]. Therefore, we believe that differentiation of human ESCs into functional thyroid follicular cells requires different *in vitro* approaches that are yet to be revealed.

## 2. Methods

### 2.1. Cell Lines and Cell Culture

Human ESCs (H9 cell line, WiCell, Madison, WI, passage 35–40) were maintained on feeder-independent culture conditions, using BD Matrigel human ESC-qualified Matrix (BD Biosciences, San Jose, CA), and grown in mTeSR-1 (Stemcell Technologies, Vancouver, Canada) at 37°C and 5% CO_2_. Cell cultures were maintained and expanded according to manufacturer's protocol. Medium was changed every day and cells were passaged using Collagenase IV (Invitrogen, Carlsbad, CA) every 6-7 days. During cell passaging only phenotypically uniform pluripotent human ESC colonies were collected. In all our experiments, we did not allow cell to go above 70% confluence (this concerns cells grow as a single layer). The human ESCs were allowed to grow in colonies such that they did not form multiple layers and did not touch each other.

### 2.2. Directed Differentiation of hESC into Definitive Endoderm

H9 human ESCs were seeded onto 6-well plates covered with BD Matrigel human ESC-qualified Matrix (BD Biosciences) at 10^5^ cells per well. Then, the cells were maintained in mTeSR1 medium at 5% CO_2_ and 37°C for two days with medium changed each day. Starting from day 3, cells in culture were maintained in differentiation media: Protocol 1, DMEM/F12 (Stemcell Technologies) supplemented with 5% KSR (knockout serum replacement) (Invitrogen), 100 ng/mL Activin A (Stemcell Technologies), 4 ng/mL bFGF), [8], and Protocol 2, DMEM/F12 supplemented with 20% KSR, 100 ng/mL Activin A, 4 ng/mL bFGF, and 20 *μ*M LY294002 (Cayman Chemical Company, Ann Arbor, MI) [9].

The differentiation medium was changed every day [9]. On the 4th day of differentiation cells were trypsinized and collected for further studies.

### 2.3. Embryoid Body Formation

 After 5 days human ESC colonies were transferred to ultra-low attachment Tx25 flasks (Corning, Corning, NY) and maintained in embryoid body (EB) media, consisting of DMEM/F12 (Stemcell Technologies) plus 15% KSR and 5% KFBS (knockout fetal bovine serum), which was changed every 48 hours. We incubated these cells for 5 days at 5% CO_2_ and 37°C to allow EBs to form.

### 2.4. Directed Differentiation to Thyroid Line

 Successful thyroid differentiation has been shown with mouse ESCs [5, 6]. We used the mouse ESCs protocol based on TSH stimulation. The following precursor cells were used for thyroid differentiation—human ESCs, embryoid bodies, and definitive endoderm cells (derived from the two protocols described previously). Cells were maintained in either of three types of thyroid differentiation media: (1) DMEM/F12 supplemented with 20% KSR, 100 mcU/mL human recombinant TSH (Fitzerald Industries international, Concord, MA): (2) DMEM/F12 supplemented with 5% FBS, 1 mU/mL bovine TSH (Sigma, St. Louis, MO): (3) DMEM/F12 supplemented with 5% FBS, 1 mU/mL bovine TSH, 10 mcg/mL human insulin (Gibco, Grand Island, NY), 6 mcg/mL transferrin (Invitrogen), 10^−8 ^M hydrocortisone (Calbiochem, Gibbstown, NJ).

On days 5, 14, and 21 cells were collected for thyroid marker expression analysis.

### 2.5. Quantitative RT-PCR

 Cells were trypsinized, washed two times with PBS, and suspended in PBS with a concentration 10,000 cells per 1 *μ*L. cDNA was synthesized using SuperScript III Cells Direct cDNA System (Invitrogen) according to manufacturer's recommendations. Quantitative real-time PCR (qPCR) was performed on iCycler iQ machine (Bio-Rad, Hercules, CA) using SYBR GreenER qPCR SuperMix for iCycler (Invitrogen). Primers were purchased from Qiagen (Valencia, CA). PCR protocol consisted of 50°C for 2 min, 95°C for 10 min, followed by 50 cycles (95°C, 15 seconds, 55°C, 30 seconds, 72°C, 30 seconds) according to Quantitect Primer Assay recommendations. Ct (cycle threshold) values were obtained for each sample, averaged over triplicates, and normalized to beta-actin, according to the formula *E* = 2^Ct  [beta−actin]−Ct  [studied  gene]^, where *E* is a normalized expression of studied gene, Ct (beta-actin) and Ct (studied gene) are Ct values of beta-actin and studied gene in corresponding samples. Normal human thyroid tissue (obtained from Laboratory of Pathology for Human Biological Materials, NIH) was used as positive control for quantitative RT-PCR. RNA was isolated from tissue with RNAqueous-4PCR kit (Ambion, Austin TX) according to manufacturer's recommendations. Obtained mRNA was converted to cDNA and processed by PCR in the same manner as with cultured cells (see above). PCR products were run on 6% precast native polyacrylamide TBE gels (Invitrogen) and stained with Ethidium Bromide (Invitrogen). DNA ladder (MassRuler, DNA Ladder Low Range, Invitrogen) was run as well and the gel images corresponding to the positions of the fragments with amplicon length of expected PCR-products were cut and presented.

### 2.6. Immunocytochemistry

For immunohistochemistry cells were grown on glass-bottom LabTek two-well Chamber Slide (BD Biosciences) in the feeder-free conditions described previously. The cell cultures were fixed with 4% paraformaldehyde for 10 minutes and then permeabilized with 0.1% Triton-X-100 in phosphate-buffered saline (PBS) for 5 minutes. Primary mouse antibodies were applied for 1 hour, and appropriately goat anti-mouse DyLight 649 labeled secondary antibodies (Abcam, Cambridge, MA) were used for 1 hour. All secondary antibodies were tested for nonspecific immunoreactivity. The following primary antibodies were used: TSHR, TPO, and NIS (Abcam). DAPI stain was used to identify the cell nuclei. After mounting in antifade media (VectaShield, Vector Laboratories, Inc., Burlingame, CA), the samples were examined by Axioplan Zeiss epifluorescent microscope (Carl Zeiss, Thornwood, NY). The camera exposure time and microscope settings were kept constant across all corresponding samples.

### 2.7. Iodine-125 Uptake Studies

Uptake of ^125^I was measured as previously described [10]. Briefly, cells were cultured in differentiation medium; after aspirating the culture medium, the cells were washed with 1 mL of Hank's balanced salt solution (HBSS) (Grocell, Manassas, VA). ^125^I uptake was initiated by adding to each well 500 *μ*L buffered HBSS containing 0.1 *μ*Ci carrier-free Na^125^I and 10 *μ*M NaI to obtain a specific activity of 20 mCi/mmol. After 1 hour at 37°C in a humid atmosphere, the radioactive medium was aspirated and cells were washed with 1 mL of ice-cold HBSS. Cells were detached by cell scraper. One mL of 95% ethanol was added to each well for 20 min and then transferred into vials for counting with a gamma counter (Biotracers, Fairfax, VA). Iodide uptake was expressed as decay per minute (dpm) per cell.

## 3. Results

### 3.1. Design of Differentiation Protocols

**Figure 1:**
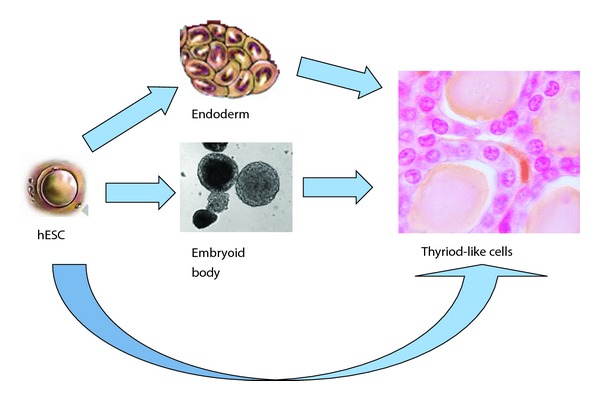
Proposed patterns of directed differentiation of human embryonic stem cells to thyroid cell line.

To achieve the differentiation of human ESCs to thyroid cells, we tried several approaches. We started with the design previously used for differentiation of mouse ESCs into thyroid cells [5, 6]. In these studies, Arufe et al. used TSH as a main differentiating factor. At the same time, leukemia-inhibiting factor (LIF), an essential component to maintain pluripotency of mouse embryonic stem cells, was removed from the medium. To obtain murine endodermal cells, they produced EBs prior to TSH stimulation. We designed modified one-step and two-step differentiation protocols for our human ESCs. First, we decided to stimulate human ESCs with TSH avoiding endoderm formation, as it was shown that in some cases, this simple approach allows for the differentiation of ESCs, for example, into osteocytes [11]. Next, we tried several approaches to obtain endodermal cells prior to stimulation with TSH. EB formation and directed differentiation to definitive endoderm were performed. Our detailed protocols are listed in Section 2. A schematic presentation of these differentiation strategies is shown in Figure 1.

### 3.2. Embryoid Body Formation

To obtain a source of endoderm cells for differentiation to thyroid cells, we formed EBs from human ESCs. EBs are three-dimensional spheroid cellular complexes, which are formed when colonies of embryonic stem cells are detached from the culture plate surface and allowed to float in the culture medium. Another important requirement for EB formation is that culture medium should be depleted of essential factors for maintaining the pluripotency of ESCs. For mouse ESCs, the essential factor is LIF, while bFGF and multiple factors produced by mouse embryonic fibroblasts (MEFs) or sold in a proprietary formulation by companies (like Stem Cells, etc.) are essential for human ESCs. Formation and morphology of EBs have been well described and their morphology was characterized in the literature [12–14]. EBs derived from human ESCs are usually allowed to grow 5–10 days, so that they become bigger in size and cell number. At this time, the morphology of formed cell complexes should be similar to the gastrulation stage of the embryo; that is, cells of three germ layers (ectoderm, mesoderm, and endoderm) should be present. Cells at this stage are often used for differentiation to more mature cell types.

**Figure 2:**
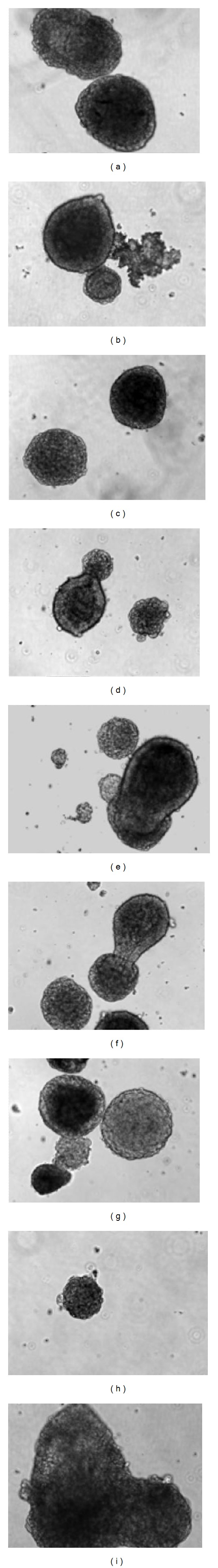
Embryoid body formation. Embryoid bodies tend to form structures similar to those seen in normal embryonic development (yolk sac, amniotic sac, etc.). The embryoid bodies vary in shape, size, and density.

**Figure 3:**
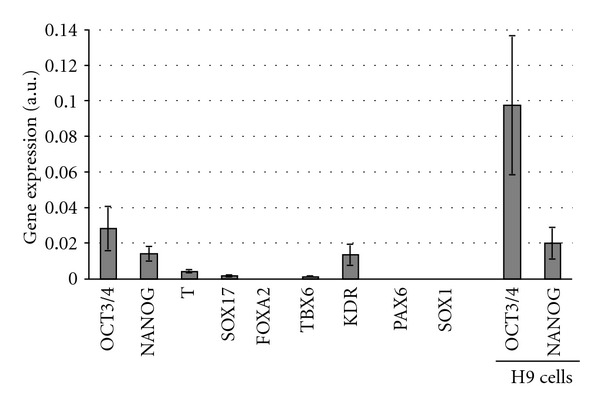
Expression of pluripotency and early differentiation mRNA markers in EBs and pluripotency markers in H9 hESCs on the right. Detailed marker description is presented in Table 1.

We formed EBs from colonies of human ESCs by allowing EBs to grow in medium that was depleted of any essential factors for 5 days (detailed protocol can be found in Section 2). Pictures of obtained EBs are shown in Figure 2. Using quantitative RT-PCR, we next measured gene expression in cells from the EBs. Positive gene markers represent early differentiation markers of all three germ layers, as well as pluripotency markers (see Table 1). The results of gene-expression studies are shown in Figure 3. As can be seen EBs express markers of all three germ layers. It is interesting to note that expression of OCT3 and NANOG is preserved. SOX17 and FOXA2 signaling for endoderm are noticeably expressed on the 5th day of EB formation, which makes cells derived from EBs a reasonable source for further differentiation to endoderm derivatives.

### 3.3. Directed Differentiation of Human ESCs to Definitive Endoderm

**Figure 4:**
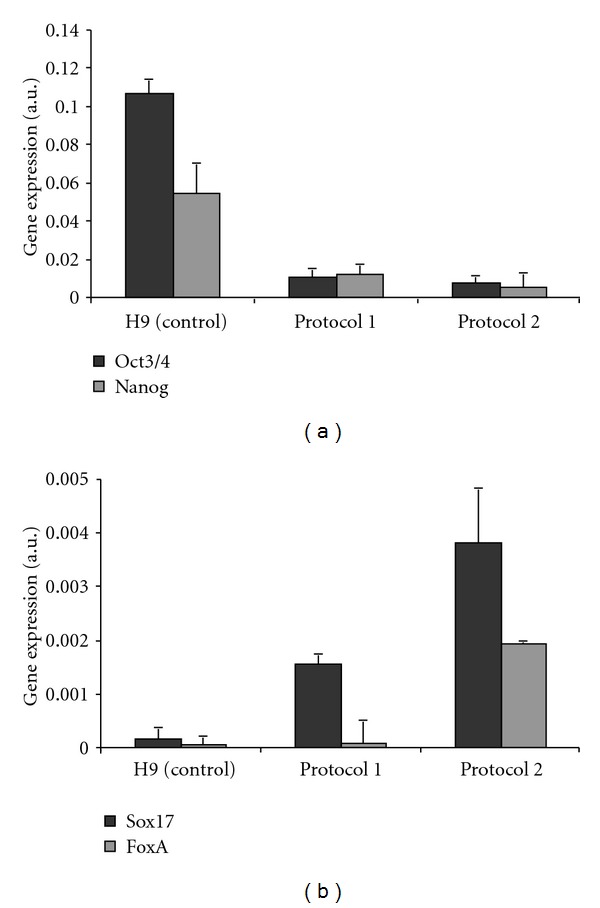
Expression of pluripotency (a) and definitive endoderm (b) mRNA markers at directed endoderm differentiation. Human ESCs were differentiated using two protocols described in Section 2. Detailed marker description is presented in Table 1.

To obtain a cell population with a high concentration of endoderm cells, we used protocols for directed differentiation of human ESCs to definitive endoderm. Two protocols from recent studies were tested. In the first approach, [8] Activin A (100 ng/mL) and human b-FGF (4 ng/mL) was used as the main differentiating factors. In the second protocol, [9] an inhibitor of PI3 kinase, LY294002, in combination with Activin A (100 ng/mL) and human b-FGF (4 ng/mL) were used. According to other authors, this last approach provides a much higher endoderm yield [9]. However, certain concerns about the differentiation capabilities of derived cells existed. We performed a gene expression assay to study pluripotency markers (OCT3, NANOG) and definitive endoderm markers (SOX17, FOXA2). The results for both protocols are presented in Figure 4. After 4 days of directed differentiation to definitive endoderm, the expression of pluripotency markers went down (Figure 4(a)), and the expression of endodermal markers increased (Figure 4(b)). In addition, the protocol implementing LY294002 appeared to be the most efficient.

### 3.4. Directed Differentiation to Thyroid-Like Cells

**Figure 5:**
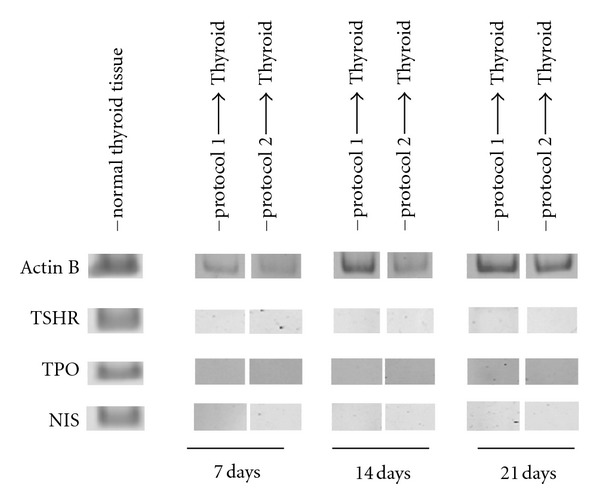
PCR results for thyroid markers expression in derived cells under different protocols and normal human thyroid tissue.

After obtaining definitive endoderm cells, we proceeded with directed differentiation to thyroid follicular cells. The following cell sources were used: human ESCs, EBs, and cells derived from directed differentiation to definitive endoderm with two protocols. Although for directed differentiation to any mature cell line it is recommended to perform step-by-step differentiation (i.e., germ layer-organ specific–tissue specific), we attempted to perform direct differentiation from human ESCs to thyroid follicular cells as well. TSH was used as a main differentiating factor because of the success of this approach for the differentiation of mouse ESC [5, 6]. Time points of 5, 14, and 21 days were chosen. First we incubated cells with 100 mcU/mL of TSH. The following thyroid-specific markers were used: TSHR (receptor to TSH), TPO (thyroperoxidase) and NIS (sodium-iodine symporter). As a positive control for quantitative RT-PCR, we used normal human thyroid tissue, which revealed expressions of all studied thyroid markers. However, expression of thyroid markers was not observed at any time point in the differentiation for all protocols tested (Figure 5). It is interesting to note that although we did not find specific bands corresponding to thyroid markers after performing gel-electrophoresis of RT-PCR products, on the 21^st^ day of differentiation in cells derived from both endoderm sources for TSHR and NIS markers, we observed PCR products of different than expected lengths. The origin of these fragments is unclear yet and will be a subject of further investigation.

 It is known that it is extremely hard to culture normal thyroid follicular cells; they survive few passages and phenotypically degrade in culture [15]. In light of this, Penna-Martinez et al. [15] suggested a protocol allowing comparatively prolonged cultivation of thyroid cells in culture obtained from fine needle aspiration biopsy (FNAB). They found a combination of defined factors that makes it possible to culture thyroid cells for up to 3 weeks with simultaneous preservation of their morphological and functional characteristics. We implemented the same strategy in our experiments adding the combination of factors to the media, as recommended by Penna-Martinez et al. [15]. As before, human ESCs, EB cells, and endodermal cells were used for differentiation. However, we still were unable to see any significant expression of thyroid markers along the differentiation pathway with this modified protocol.

Additionally, we performed immunocytochemistry studies with cells on the 21^st^ day of directed thyroid differentiation. We assumed that a small population of derived cells might possess thyroid features. However, none of the tested markers (TSHR, TPO, NIS) appeared to be positive (data not shown).

### 3.5. ^125^I Uptake Studies

We performed a functional assay to establish the ability of differentiated cells to take up iodine. Even though we could not detect a significant expression of the sodium-iodine symporter, we proceeded with radioactive iodine studies as a sensitive functional assay. We incubated cells with a mixture of radioactive and cold iodine according to a well-established protocol [10] and measured intracellular ^125^I with gamma-counter after 1 hour of incubation. We measured iodine uptake in “thyroid like” cells derived from the different studied protocols; however, we did not observe a statistically significant increase in radioiodine uptake under any of these experimental conditions.

## 4. Discussion

The sodium iodine symporter has been an important subject of nuclear medicine research for decades, because it serves as an effector for cell-localized radiation therapy due to its unique ability to concentrate iodine inside a cell. Iodine-131 radiotherapy in clinical practice causes the results of thyroid cancer treatment to improve dramatically. But several important issues still need to be resolved, for example, the treatment of anaplastic thyroid cancer, which lacks NIS expression in its cellular membranes. Also, other tissues like mammary glands are known to express low levels of NIS [16–18], which potentially could be used for breast cancer treatment [18–23]. A better understanding of the appearance of NIS during embryologic development may help to explain why fetuses of children are so sensitive to medical and environmental radioactive exposures. The structure of NIS and its regulation are well studied, and numerous approaches to induce or improve its functioning have been proposed from gene transfection [18, 24–26] to pharmacological induction [27]. However, many of them lack the potential for clinical translation. The hypothesis we proposed used human embryonic stem cells as a model for studying NIS maturation at different stages of differentiation towards thyroid follicular cells. This approach might provide insight into the mechanism of NIS regulation in cancer dedifferentiation which is opposite in its apparent expression of NIS.

In this study, we attempted to develop the protocol for directed differentiation of human ESCs to thyroid cells. A similar approach has been successfully used by Arufe et al. [5] in mouse ESCs. They obtained “thyroid-like cells” possessing features of thyroid cells. The derived cells expressed main thyroid markers (TSHR, NIS, TPO, TG, PAX8) determined by RT-PCR and western blot. Arufe et al. [5] proposed a two-step protocol for mouse ESCs: and Step 1, obtaining endoderm cells through generation of EBs; Step 2, stimulation of EB cells by TSH to derive thyroid cells. In our study, we used a similar approach; in addition, several modifications and variations were implemented. We used H9 human embryonic stem cell line as one of the most studied cell lines and proved to be a good source for successful differentiation to variety of cell types In our experiments, we were able to obtain human endoderm cells using three different protocols: (1) using EB formation as in the mouse ESCs study described above: (2) using definitive factors (Activin A, bFGF), and (3) LY294002, PI3-kinase (phosphoinositide 3-kinase) inhibitor for directed endoderm formation [8, 9].

However, concerns exist regarding the potential of derived endoderm cells to form more specialized and mature cell types when these protocols are used; especially with LY294002 as PI3-kinase participates in many pathways and its inhibition may affect differentiation abilities of the cells. Nevertheless, several studies showed successful differentiation of endodermal cells into liver [28–30], pancreatic, and lung cells [30, 31]. We used endoderm cell derived from all three protocols for further differentiation into the thyroid line. TSH was used as a main differentiation factor. Since gestation period is longer in humans compared to mice, we allowed cells to grow longer (up to 21 days). We also varied the concentration of TSH and did not observe any clear expression of thyroid markers in any of the group of cells. A possible explanation for the absence of observable thyroid markers was that cells might differentiate to thyroid cells but could not survive in the culture conditions. We then acquired the previously proposed protocol for culturing of thyroid cells obtained from a fine needle aspiration biopsy [15]. This protocol maintained thyroid cells in culture up for to 3 weeks. However, adding essential factors from this protocol to our differentiation media did not result in the formation of cells possessing thyroid features. We also measured iodine uptake by cells derived with different protocols using ^125^I. We did not see any changes between control and experimental cells.

We think that even though differentiation pathways in mice and humans might possess similar features, many molecular mechanisms may vary. This also concerns source cells used for differentiation. Even though it is claimed that mouse ESCs used by Arufe et al. [5, 6] and H9 cells were taken from inner cell mass of blastocysts, concerns may arise about the terms of blastocyst stage as in early embryogenesis cells differentiate and maturate extremely fast, such that mere hours may matter. Also it was shown [32] that even within one species ESCs might vary in their biological properties, namely, in their ability to differentiate to various tissues, if they belong to different cell lines, or if they are of different passages even within the same cell line. Besides, there is another important point which might explain observed differences. In experiments with mouse ESCs [5, 6] and in our experiments along the differentiation cells were growing as a monolayer (on Matrigel for human ESCs). It is possible that 2D geometry can be appropriate for differentiation towards thyroid line for mouse ESCs, while for human ESCs spatial orientation of cells might be a strong requirement even if stimulation with essential factors is correct. In this case 3D cell culture method must be attempted. One of the limitations of this study is using only one human embryonic cell line, H9. Even though cells from this cell line were successfully differentiated toward many cell types, including endoderm derivatives, we still have to admit that luck of differentiation to thyroid cells might be related to peculiarities of H9 cell line, and thus, results may not generalizable to all human embryonic cell lines.

## 5. Conclusions

Based on the results of our experiments, we conclude that differentiation of H9 human ESCs into thyroid cells cannot be induced by TSH media supplementation alone and most likely involves complicated developmental patterns that are yet to be determined.

## Figures and Tables

**Table 1:** Markers and their description indicating pluripotency, three germ layers, and thyroid tissue.

Pluripotency	Ability to give rise to all tissues of the body	Oct3/4	POU class 5 homeobox 1
		Nanog	Nanog homeobox
Ectoderm	Nervous system, neural crest and its derivatives, epidermis, eye, salivary, and skin glands	PAX6	Paired box 6
		SOX1	SRY-box 1
Mesoendoderm	Gives rise to mesoderm and endoderm	T	T, brachyury homolog
Mesoderm	Connective tissue, muscles, reproductive system, spleen, peritoneum, urinary system, mesothelium, heart	TBX6	T-box 6
		KDR	kinase insert domain receptor
Definitive endoderm	Digestive and respiratory tracts, endocrine glands, and organs such as the liver and pancreas	SOX17	SRY-box 17
		FOXA2	Forkhead box A2
Thyroid tissue	Thyrocytes within thyroid gland derived from definitive endoderm	TSHR	Receptor to TSH
		TPO	Thyroperoxidase
		NIS	Sodium-iodine symporter

## Abbreviations

ESCs: Embryonic stem cells
EBs: Embryoid bodies
TSH: Thyroid-stimulating hormone
NIS: Sodium-iodine symporter
TSHR: Thyroid-stimulating hormone receptor
TPO: Thyroid peroxidase
TG: Thyroglobulin
MEFs: Mouse embryonic fibroblasts
LIF: Leukemia-inhibiting factor
PI3-kinase: Phosphoinositide 3-kinase.
